# Optimizing C14120-based lipid nanoparticles for mRNA delivery: Editorial commentary

**DOI:** 10.1016/j.omtn.2026.102945

**Published:** 2026-05-25

**Authors:** Vincent Fung, Caroline Argenti, Maggie Magaw, Owen S. Fenton

**Affiliations:** 1 Division of Pharmacoengineering and Molecular Pharmaceutics, Eshelman School of Pharmacy, University of North Carolina at Chapel Hill, Chapel Hill, NC 27599, USA

## Main text

The pursuit of developing nanomedicines capable of extrahepatic delivery has driven rapid growth in new nanomaterials and nanoparticle formulations.^1^^,^^2^^,^^3^^,^^4^^,^^5^ Thus, developing a rational and effective strategy to rapidly screen and down-select these newly developed formulations for their overall delivery performance *in vitro* and *in vivo* could be beneficial to the nanomedicine field. In this issue of *Molecular Therapy Nucleic Acids*, Song et al. developed a workflow using the ionizable lipid C14120 to generate a library of lipid nanoparticles (LNPs) with diverse formulation ratios. They applied an *in vivo* DNA barcoding strategy to rapidly screen, identify, and down-select formulations with high potential for extrahepatic delivery.^6^

Several of the recent advancements in the nanomedicine field may include the development of customizable nanoparticle platforms that deliver nucleic acid payloads of various modalities, such as plasmid DNA, mRNA, CRISPR-Cas9, and siRNA, to various organs and cell types of interest in addition to the liver.^1^^,^^2^^,^^3^^,^^4^^,^^5^^,^^7^ Building on these platforms, the nanomedicine field has broadly investigated the interactions between nanoparticles and their cellular targets, which may suggest a wide range of mechanisms that include protein corona, endosomal escape, and cellular uptake.^1^^,^^2^^,^^3^^,^^4^^,^^5^^,^^8^ Leveraging this, future development of nanomaterials and nanoparticle formulations, when coupled with high-throughput screening, may assist in refining the nanomedicine’s design, performance, and safety profile.

The authors first created 25 mRNA-LNPs with the C14120 ionizable lipid, cholesterol, helper lipids (dioleoylphosphatidylethanolamine [DOPE] or dioleoylphosphatidylcholine [DOPC]), and PEG-lipids (D-ɑ-tocopherol polyethylene glycol succinate [TPGS] or 1,2-distearoyl-sn-glycero-3-phosphorylethanolamine [DSPE]-polyethylene glycol [PEG]-2000), which were formulated with varying component molar ratios. The following trends were observed: (1) mRNA-LNP sizes generally increased as the concentration of helper lipids increased, (2) mRNA-LNP sizes generally increased as the concentration of C14120 ionizable lipids increased, (3) mRNA-LNP sizes generally decreased as the concentration of PEG-lipids increased, (4) polydispersity (PDI) of mRNA-LNPs was mostly below 0.3, (5) mRNA-LNP sizes were generally kept within 100–200 nm, (6) mRNA-LNP size was smallest when TPGS PEG-lipid composition was kept at 14%, (7) mRNA encapsulation was generally above 75% when a N/P ratio of 12 was kept, and (8) mRNA-LNP zeta potentials were generally kept within 30–70 mV.^6^

Encouraged by the preliminary results, the authors performed *in vitro* studies using the library of mCherry mRNA-LNPs (LNPs 1 to 12 contain TPGS, while LNPs 13 to 24 contain DSPE-PEG-2000) on various cell lines (e.g., ARPE-19 human retinal pigment epithelial cells, HepG2 human hepatocellular carcinoma cells, AML12 mouse hepatocytes, and HEK293 human embryonic kidney cells) to investigate the effect of helper lipid (DOPE or DOPC) and PEG-lipid (TPGS or DSPE-PEG-2000) composition. The following general trends emerged: (1) in ARPE-19 and AML12 cell lines, irrespective of the choice or absence of helper lipid, the mCherry MFI generally decreased as the concentration of either PEG-lipid increased; (2) in HepG2 cells, the mCherry MFI generally increased as the concentration of TPGS PEG-lipid increased; and (3) in HEK293 cells, the mCherry MFI generally decreased as the concentration of TPGS PEG-lipid increased for mRNA-LNPs that had DOPC in their formulation, while mCherry MFI generally increased when DOPE or no helper lipid was used as TPGS PEG-lipid increased.^6^ The findings suggest that certain cell lines may prefer certain LNP formulations perhaps by differences in formulation components and their compositions, perhaps highlighting the tunability of the LNP platform for future applications.

Following the *in vitro* cell transfection results with the mCherry mRNA-LNP library, the authors utilized the DNA barcoding system to rapidly screen for suitable mRNA-LNP library members that had desirable targeting, uptake, and transfection efficiency *in vitro* as well as *in vivo*.^9^ In brief, a unique 58-nt long ATTO-DNA barcode sequence (barcode-ATTO647) was co-encapsulated in individual mRNA-LNPs at a 10:1 (mRNA:DNA) weight ratio prior to delivery. By detecting the fluorescence signal from ATTO-DNA barcode sequence, the authors could quantify the cellular uptake of mRNA-DNA-LNPs and the protein expression from the mRNA payload at the same time. The *in vitro* DNA barcoding validation experiment may suggest that the uptake (ATTO-DNA barcode fluorescence signal) may not correlate fully with protein expression (EYFP-mRNA fluorescence signal) across different mRNA-DNA-LNP formulations and different cell lines (AML12 and HEK293) in this study.^6^

Subsequently, the authors used the same DNA-barcoding strategy to deliver a library of mRNA-DNA-LNPs (luc-mRNA:ATTO-DNA barcode weight ratio of 10:1) to mice *in vivo* via an intravenous (i.v.) administration at a dose of 1 mg mRNA/kg mice. After 24 h, the bulk nucleic acids were extracted from various mice organs (liver, lung, spleen, heart, kidney, and brain) and were sequenced with next-generation Illumina sequencing. The authors discovered the following trends: (1) the DNA-barcode corresponding to LNP 11 was highly enriched in multiple organs (predominantly in the liver), (2) the DNA barcode in LNP 8 was highly enriched in the lungs, (3) the DNA barcode in LNP 5 was highly enriched in the brain, (4) the DNA barcode in LNP 14 was highly enriched in the spleen, (5) the relative abundances of DNA barcodes across the organ replicates corresponding to each LNP formulation remain consistent, and (6) high enrichment of DNA barcodes corresponding to LNP 11, 12, and 13.^6^

The authors subsequently validated the organ-tropic LNP candidates down-selected from the DNA-barcoding strategy by monitoring the luciferase protein expression post-i.v. administration. Interestingly, although LNP 8 had higher luciferase expression in the lungs (lung/liver = 2), which aligns with the DNA-barcoding results, the luciferase expression profiles yielded by LNP 11 and 14 may not align with the DNA-barcoding results. LNP 11 had a much higher luciferase expression in the lungs than LNP 8 (lung/liver = 5) although the DNA-barcode was enriched in the liver, while LNP 14 had negligible luciferase expression in the spleen despite having enrichment of DNA-barcode in the spleen.^6^

Building on these findings, future studies may further advance the nanomedicine research field forward by improving screening strategies. For instance, mRNA-based barcoding strategies may link delivery to functional expression, which could minimize potential confounding effects of the DNA barcode and co-loading dynamics with mRNA. The disconnect between barcode uptake and protein expression in various cell types may also underscore the complex nature of RNA delivery and the opportunities for a predictive-design framework. Future studies may also be conducted to clarify the role of C14120 ionizable lipid and the respective PEG-lipid and helper lipid component and composition ratios on extrahepatic delivery, as well as their endosomal escape properties and their affinity toward certain organ-tropic protein corona.^8^^,^^10^ For example, one of the intracellular barriers, such as endosomal escape, may vary across different cell types. Improving endosomal escape as one of the strategies to improve mRNA-LNP delivery could be achieved with further mechanistic studies to understand how lipid chemistry could influence membrane destabilization and cargo release.^8^^,^^10^ Overall, this work aims to advance both hepatic and extrahepatic targeting strategies through tuning the LNP composition. Taken collectively, this study may contribute to the advancing landscape of RNA-based therapeutics by improving the design and evaluation of targeted mRNA-LNP formulations.

## Declaration of interests

The authors declare no competing interests.
